# Führt eine COVID-19-bedingte Ausgangsbeschränkung zu einer Reduktion schwer verletzter Patienten an einem überregionalen Traumazentrum?

**DOI:** 10.1007/s00113-020-00924-1

**Published:** 2020-11-30

**Authors:** Konrad F. Fuchs, Lars Eden, Fabian Gilbert, Silvia Bernuth, Thomas Wurmb, Rainer H. Meffert, Martin C. Jordan

**Affiliations:** 1 Klinik und Poliklinik für Unfall‑, Hand‑, Plastische und Wiederherstellungschirurgie, Universitätsklinikum Würzburg, Oberdürrbacher Straße 6, 97080 Würzburg, Deutschland; 2 Klinik und Poliklinik für Anästhesiologie, Intensivmedizin und Schmerztherapie, Universitätsklinikum Würzburg, Oberdürrbacher Straße 6, 97080 Würzburg, Deutschland

**Keywords:** Intensivkapazitäten, Coronavirus, Injury Severity Score, Polytrauma, Pandemie, ICU capacities, Corona virus, Injury severity score, Polytrauma, Pandemic

## Abstract

**Hintergrund:**

Intensiv- und Beatmungskapazitäten sind für die Behandlung COVID-19-erkrankter Patienten essenziell. Unabhängig davon beanspruchen auch schwer verletzte Patienten häufig Intensiv- und Beatmungskapazitäten. Daraus ergibt sich folgende Fragestellung: Führt eine Ausgangsbeschränkung zu einer Reduktion schwer verletzter Patienten, und kann hierdurch mit frei werdenden Intensivkapazitäten gerechnet werden?

**Material und Methoden:**

Es erfolgte eine retrospektive Auswertung schwer verletzter Patienten mit einem Injury Severity Score (ISS) ≥16 zwischen dem 17.03.2020 und 30.04.2020 (landesweiter Shutdown) an einem überregionalen Traumazentrum. Erfasst wurden der Unfallmechanismus, ISS, Versicherungsträger (BG vs. GKV/PKV), ob es sich um einen dokumentierten Suizidversuch handelte, und ob eine operative Intervention innerhalb der ersten 24 h erforderlich war. Als Kontrollgruppe wurden die Daten des gleichen Zeitraums der Jahre 2018 und 2019 ausgewertet.

**Ergebnisse:**

Es konnte keine wesentliche Veränderung bezüglich der Anzahl an schwer verletzten Patienten festgestellt werden (2018 *n* = 30, 2019 *n* = 23, 2020 *n* = 27). Es zeigten sich insgesamt keine deutlichen Veränderungen der absoluten Zahlen bezüglich der Intensivpflichtigkeit in den ersten 24 h und der Beatmungspflichtigkeit beim Verlassen des Schockraums. Die Anzahl an Patienten, die eine Operation innerhalb der ersten 24 h nach Eintreffen im Schockraum benötigten, war 2020 sogar leicht erhöht, jedoch nicht statistisch signifikant. Der durchschnittliche ISS blieb konstant. Bezüglich der Unfallursache zeigte sich 2020 kein Motorradfahrer, der einen nicht berufsgenossenschaftlich versicherten Unfall erlitt (2018 *n* = 5, 2019 *n* = 4, 2020 *n* = 0). Es wurde 2020 ein erhöhter Anteil an Arbeitsunfällen mit einem ISS ≥16 festgestellt (2018: 10 %, 2019: 26,1 %, 2020: 44,4 %).

**Diskussion:**

Eine Ausgangsbeschränkung führte zu keiner Reduktion verletzter- und intensivpflichtiger Patienten am untersuchten Zentrum. Auch unter einer landesweiten Ausgangsbeschränkung muss für dieses Patientenkollektiv eine ausreichende Menge an Intensiv- und OP-Kapazitäten vorgehalten werden. Die Bestätigung dieser Ergebnisse durch Auswertung nationaler Register steht noch aus.

## Abstract (en)

**Background:**

Intensive care and ventilator capacities are essential for treatment of COVID-19 patients. Severely injured patients are often in continuous need of intensive care and ventilator treatment. The question arises, whether restrictions related to COVID-19 have led to a decrease in severely injured patients and thus to an increase in intensive care unit (ICU) capacity.

**Material and methods:**

A retrospective analysis of all seriously injured patients with an injury severity score (ISS) ≥16 was performed between 17 March and 30 April 2020 at a level 1 trauma center in Germany. The mechanism of injury and the ISS were recorded. Further data were collected as to whether it was a work-related accident, a documented suicide attempt and if surgery was necessary in the first 24 h after arrival in hospital. Data from 2018 and 2019 served as a control group.

**Results:**

There was no substantial difference in the total number of seriously injured patients (2018 *n* = 30, 2019 *n* = 23, 2020 *n* = 27). Furthermore, there was no relevant difference in the number of patients needing intensive care or ventilator treatment when leaving the shock room. The number of patients needing an operative intervention within the first 24 h after arriving at hospital was slightly higher in 2020. The mean ISS was at a constant level during all 3 years. In 2020 there was no polytraumatized motorbike rider, who did not have a work-related accident (2018 *n* = 5, 2019 *n* = 4, 2020 *n* = 0). A noticeable increase in work-related accidents was observed (2018: 10%, 2019: 26.1%, 2020 44.4%).

**Discussion:**

Restrictions related to COVID-19 did not lead to a reduction in seriously injured patients needing ICU care. Due to the monocentric data analysis there is room for misinterpretation.

In general, intensive care and operating capacities should be managed with adequate consideration for seriously injured patients even in times of crisis, such as the COVID-19 pandemic. Confirmation through the German Trauma Register is pending.

## Hintergrund und Fragestellung

„Corona Virus Disease“ 2019 (COVID-19) hat zu einer der größten gesundheitlichen und gesellschaftlichen Krisen der Nachkriegszeit geführt [11]. Durch die Rasanz der Ausbreitung herrschte selbst in einigen Industrienationen ein unerwarteter Versorgungsengpass an Intensiv- und Beatmungskapazitäten [9, 12]. Innerhalb kürzester Zeit mussten Krisenkonzepte zur Versorgung potenziell beatmungspflichtiger Patienten etabliert werden [10, 15]. Trotz hoher Intensivkapazität wurden in Deutschland vorsorglich zusätzliche Beatmungsbetten generiert. Das operative Elektivprogramm kam fast vollständig zum Erliegen, um vorhandene Intensivkapazitäten zu schonen [3, 8].

Gerade schwer verletzte Patienten benötigen häufig eine operative Intervention als auch eine intensivmedizinische Überwachung. Außerdem binden Schockraumeinsätze medizinisches Personal aus verschiedenen Fachrichtungen für längere Zeit. Die durchschnittliche Zeit, von Ankunft im Schockraum bis zur OP, betrug innerhalb der letzten 10 Jahre bei teilnehmenden Kliniken des Traumaregisters der deutschen Gesellschaft für Unfallchirurgie (TraumaRegister DGU®, TR-DGU) 75,7 min (±61,1 min) [14].

Im Rahmen der rapiden Entwicklungen der Pandemie wurde in Bayern am 16.03.2020 der Katastrophenfall ausgerufen [1]. Ferner wurden umfangreiche Kontakt- und Ausgangsbeschränkung ab dem 17.03.2020 festgelegt, die das alltägliche Leben grundlegend einschränkten [2]. Die Bevölkerung wurde angehalten, das Risiko von Verletzungen durch Freizeitaktivitäten so gering wie möglich zu halten, um die medizinischen Kapazitäten für die Versorgung von COVID-19-Patienten zu erhalten. In Bayern wurde Motorradfahren nur für den Dienstweg oder wichtige Botengänge gestattet. Des Weiteren wurde ausgewählten Berufsgruppen das Fortführen ihrer Beschäftigung untersagt [2].

Die Dauer und der Verlauf der aktuellen als auch zukünftiger Pandemien lassen sich nicht absehen. Zur besseren Planbarkeit von OP- und Intensivkapazitäten stellt sich daher die Frage, wie sich die Ausgangsbeschränkung auf die Anzahl der schwer verletzten Patienten, die Art des Unfallgeschehens und die OP-Notwendigkeit an einem überregionalen Traumazentrum in Bayern auswirkt.

## Material und Methoden

### Datenerhebung- und Aufarbeitung

Es erfolgte eine retrospektive Auswertung der Daten des Universitätsklinikums Würzburg (UKW) für den Zeitraum vom 17.03. bis 30.04. der Jahre 2018, 2019 und 2020. Das UKW zählte mit 241 schwer verletzten Patienten (Maximum Abbreviated Injury Score ≥3) im Jahr 2018 zu den überregionalen Traumazentren mit den höchsten Fallzahlen in Deutschland [14]. Der ISS der eingelieferten Patienten wurde bestimmt und alle Verletzten mit einer Punktzahl ≥16 in diese Studie eingeschlossen. Der ISS ist ein Instrument, mit dem die Gesamtverletzungsschwere objektiviert werden kann. Er kann einen Wert von 1–75 annehmen und berechnet sich aus dem Quadrat des Abbreviated-Injury-Scale-Werts (AIS) der 3 am schwersten verletzten Körperregionen [4]. Erfasst wurden ferner der Unfallmechanismus (unterschieden wurde zwischen Fahrrad- und Fußgängerunfällen, körperlicher Gewalt, Unfällen durch landwirtschaftliche Maschinen, LKW-, Motorrad- und PKW-Unfällen, Quetschungen, Stürzen, Schussverletzungen, Überrolltraumata und Verletzungen durch herabfallende Teile), ob es sich um einen Arbeits- oder Wegeunfall handelte, ob es sich um einen dokumentierten Suizidversuch handelte, ob eine Intensivpflichtigkeit in den ersten 24 h bestand, ob der Patient beim Verlassen des Schockraums intubiert und ob eine Operation innerhalb der ersten 24 h nach Trauma erforderlich war.

### Datenanalyse und Statistik

Es erfolgte eine deskriptive Statistik und ein Test auf Normalverteilung (Kolmogorov-Smirnov- und Shapiro-Wilk-Test). Die nominalen Daten wurden anhand des Chi-Quadrat-Tests auf signifikante Unterschiede überprüft. Beim nichtnormalverteilten ISS erfolgte anschließend ein Kruskal-Wallace-Test. Das Signifikanzniveau wurde auf *p* < 0,05 festgesetzt.

## Ergebnisse

**Figure 1:**
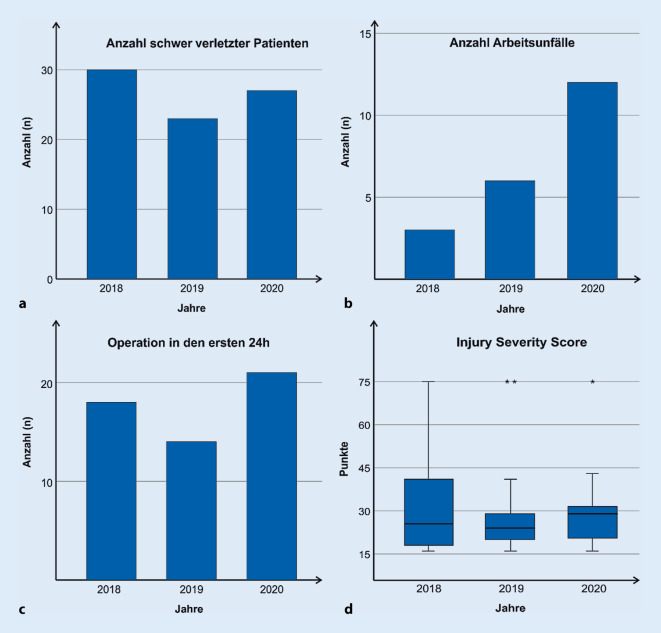

**Figure 2:**
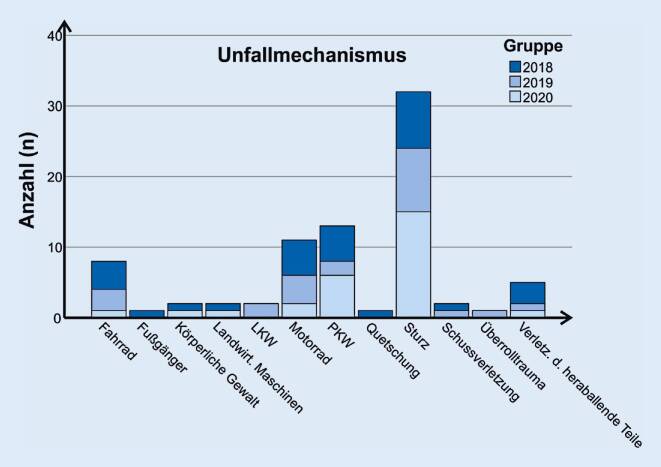

Die Gesamtanzahl an schwer verletzten Patienten mit einem ISS ≥16 betrug im Zeitraum vom 17.03. bis zum 30.04. im Jahr 2018 *n* = 30, im Jahr 2019 *n* = 23 und im Jahr 2020 *n* = 27 (Abb. 1a). Es zeigte sich ein signifikanter Unterschied (*p* *=* *0,013*) bei der Anzahl der Arbeits- und Wegeunfälle (2018 *n* = 3, 2019 *n* = 6, 2020 *n* = 12; Abb. 1b). Im festgelegten Zeitraum kam es 2020 zur Versorgung von 2 schwer verletzten Motorradfahrern (im Vergleich: 2018 *n* = 5; 2019 *n* = 4). Im Jahr 2020 handelte es sich bei den verletzten Motorradfahrern ausschließlich um Wegeunfälle, während die polytraumatisierten Motorradfahrer in den beiden Vorjahren in ihrer Freizeit verunfallten. In allen Jahren war der Unfallmechanismus „Sturz“ führende Ursache des Polytraumas (Abb. 2). Die Anzahl dokumentierter Suizidversuche mit Einweisung in den Schockraum im Kollektiv 2020 war leicht erhöht (2018 *n* = 1, 2019 *n* = 2, 2020 *n* = 4), jedoch nicht signifikant (*p* = 0,309). Der durchschnittliche ISS lag 2018 bei 32 (SD ± 19), 2019 bei 28 (SD ± 16) und 2020 bei 29 (SD ± 12) Punkten (Abb. 1d). Es zeigten sich diesbezüglich keine statistisch signifikanten Unterschiede (*p* = 0,767). Alle Patienten benötigten eine Intensiv- oder IMC-Überwachung in den ersten 24 h nach Trauma. Beim Verlassen des Schockraums waren 2018 *n* = 22, 2019 *n* = 18 und 2020 *n* = 19 Patienten beatmet. Die Unterschiede waren nicht signifikant (*p* = 0,817). Die Notwendigkeit eines operativen Eingriffs innerhalb der ersten 24 h ist im Jahr 2020 im Vergleich zu den Vorjahren leicht gestiegen (2018 *n* = 18, 2019 *n* = 14, 2020 *n* = 21), es konnte jedoch kein statistisch signifikanter Unterschied (*p* = 0,297) festgestellt werden (Abb. 1c).

## Diskussion

Es handelt sich bei der COVID-19-Pandemie um eine Krise mit schwer kalkulierbarem Verlauf. Bisherige Entscheidungen mussten häufig ohne verlässliche Informationen über den Krankheitserreger oder die Versorgungsstrategie getroffen werden.

Die vorliegenden Daten beziehen sich auf einen sehr kurzen Zeitraum, und allgemeine Rückschlüsse lassen sich aufgrund starker Limitationen der Studie nicht mit Sicherheit ziehen. Eine Ausgangsbeschränkung in diesem Ausmaß ist in der Nachkriegsgeschichte jedoch einmalig, und es liegen keine vergleichbaren Daten vor. Für aussagekräftigere Daten ist eine landesweite Auswertung, wie z. B. anhand des TR-DGU, dringend erforderlich. Diese ist jedoch zeitintensiv und wird im Rahmen des TR-DGU voraussichtlich erst Mitte 2021 möglich sein. Aufgrund der Aktualität der Situation und einer zweiten Infektionswelle durch COVID-19 sind die vorliegenden Daten aber möglicherweise wertvoll zur weiteren Planung der Intensivkapazitäten und Koordinierung des Schockraumbedarfs.

Am UKW konnte keine wesentliche Reduktion der Anzahl schwer verletzter Patienten festgestellt werden. Die Notwendigkeit einer operativen Intervention innerhalb der ersten 24 h nach Ankunft im Schockraum ist sogar leicht gestiegen. Dementsprechend muss im ungünstigsten Fall davon ausgegangen werden, dass durch die Ausgangsbeschränkung nicht automatisch mit frei werdenden räumlichen, materiellen oder personellen Ressourcen aus der Schwerverletztenversorgung gerechnet werden kann. Vielmehr muss von einer konstanten Fallzahl unfallchirurgischer Patienten ausgegangen werden. Von Maßnahmen, wie z. B. Kurzarbeit oder Homeoffice, sollte in unfallchirurgischen Schwerpunktkliniken mit hohem Notfallaufkommen daher auch während solcher Krisensituationen abgesehen werden.

Bezüglich des Unfallmechanismus zeigte sich ein Rückgang an „Freizeitunfällen“ im Vergleich zu den letzten beiden Jahren. Erstaunlicherweise wurde ferner eine deutlich erhöhte Anzahl Schwerverletzter durch Wege- und Arbeitsunfälle festgestellt. Ein möglicher Grund für die relative Zunahme an Schwerverletzten durch Arbeits- und Wegeunfällen könnte evtl. durch eine Zentralisierung der Schwerverletztenversorgung während des landesweiten Shutdown bedingt sein. Möglicherweise wurden in dieser Zeit Schwerverletzte primär in überregionale Traumazentren verbracht, um regionale Krankenhäuser zu entlasten. Auch in diesem Punkt ist eine Auswertung nationaler Register abzuwarten.

Eventuell ist dies auch durch ein konsequentes Einhalten der Maßnahmen der Infektionsschutzmaßnahmenverordnung zu werten.

Dafür sprechen zusätzlich die Daten an polytraumatisierten Motorradfahrern. Im April 2020 gab es in Bayern insgesamt 295 Sonnenstunden, 2019 215 Sonnenstunden und 2018 250 Sonnenstunden [5–7]. Es kam, trotz des guten Wetters, zu keinem polytraumatisierten „Freizeitmotorradfahrer“ im gesamten Zeitraum des Shutdowns. Ob sich die Bevölkerung an das Verbot des Motorradfahrens zur Freizeitgestaltung gehalten hat, lässt sich durch unsere Daten zwar nicht zweifelsfrei belegen, aber zumindest kam es zu keinem relevanten Unfall.

Aufgrund des geringeren Pendler- und Berufsverkehrs war eine verringerte Anzahl an schwer verletzten PKW-Fahrern zu erwarten. Die Daten des Statistischen Bundesamtes ergaben im März 2020 eine Reduktion der Verkehrsunfälle um 23 % im Vergleich zum Vorjahr [13]. Die Zahl der Verkehrstoten lag mit 158 sogar auf dem tiefsten Stand seit 1990 [13]. Die am UKW erhobenen Daten bestätigen dies jedoch nicht uneingeschränkt.

Die geringe, statistisch nichtsignifikante Zunahme an Suizidversuchen in dem beobachteten Patientenkollektiv während der Ausgangsbeschränkung im Vergleich zu den Vorjahren lässt aufgrund des kleinen Patientenkollektivs keine allgemeinen Rückschlüsse zu.

### Limitationen

Die monozentrische Datenerhebung birgt das große Risiko der Fehlinterpretation. Möglicherweise verursachte ein geringeres Patientenaufkommen in lokalen und regionalen Traumazentren die konstant hohe Zahl an überregionalen Kliniken. Weiterführende Datenauswertung, wie z. B. über das TR-DGU sowie durch die Daten der Krankenkassen sind deshalb dringend erforderlich. Es ist denkbar, dass die Erkenntnisse aus den nationalen Registern die hier berichteten Daten dann auch teilweise widerlegen.

## Fazit für die Praxis

- Eine Ausgangsbeschränkung führt anhand der hier erhobenen, aber limitierten Daten, tendenziell zu keiner relevanten Reduktion schwer verletzter Patienten an einem überregionalen Traumazentrum.
- Es kann nicht automatisch mit frei werdenden räumlichen, materiellen oder personellen Ressourcen aus der Polytraumaversorgung gerechnet werden.
- Zur Vermeidung eines Qualitätsverlusts sollte eine personelle und materielle Ausstattung ohne Einschränkung sichergestellt werden.
